# Effectiveness of the settings-based intervention Shaping the Social on preventing dropout from vocational education: a Danish non-randomized controlled trial

**DOI:** 10.1186/s40359-018-0258-8

**Published:** 2018-09-12

**Authors:** Susan Andersen, Morten Hulvej Rod, Teresa Holmberg, Liselotte Ingholt, Annette Kjær Ersbøll, Janne Schurmann Tolstrup

**Affiliations:** 1 Centre for Intervention Research in Health Promotion and Disease, National Institute of Public Health, University of Southern Denmark, Studiestræde 6, DK-1455 Copenhagen K, Denmark; 2 National Research Centre for Disadvantaged Children and Youth, Kronprinsesse Sofies Vej 35, Frederiksberg, Denmark; 3 National Institute of Public Health, University of Southern Denmark, Studiestræde 6, DK-1455 Copenhagen, Denmark

**Keywords:** Student dropouts, Prevention, Social environment, Wellbeing, Settings-based intervention

## Abstract

**Background:**

Lack of formal education is an important social determinant of health inequality and represents a public health problem. School dropout is particularly common in vocational education; however few prevention programs targeting dropout in the vocational school setting have been evaluated. The purpose of the present study was to test the effect on school dropout of a settings-based intervention program (named Shaping the Social) that targeted the school organization in order to create social and supportive learning environments.

**Methods:**

A non-randomized controlled design including four large intervention schools and six matched-control schools was used. The target population was students in technical and agricultural vocational education, which is provided to students from age 16. Students were enrolled at school start. Register-based data (*n* = 10,190) was used to assess the effect on school dropout during a 2-year period. Odds ratios (OR) and 95% confidence intervals (CI) were calculated in logistic regression models, adjusting for age, sex, ethnicity, parental income, prior school dropout and type of basic course. Student survey (*n* = 2396) at 10-week follow-up was used to examine wellbeing at school (four subscales: school connectedness, student support, teacher relatedness, and valuing the profession) which was the hypothesized proximal intervention effect. As a secondary aim, we examined how the student wellbeing factors were associated with school dropout, independently of the intervention, and we explored whether the student wellbeing factors were potential mediators.

**Results:**

The present study showed an intervention effect on school dropout with dropout rates lower in intervention schools (36%) than control schools (40%) (OR = 0.86, 95% CI: 0.74, 0.99). We had no attrition on the dropout outcome. School connectedness mediated the intervention effect; no significant mediation effects were found for student support, teacher relatedness, and valuing the profession. Independently of the intervention, each student wellbeing factor prevented dropout.

**Conclusions:**

Findings from this study suggest that a comprehensive, multicomponent school-based intervention could prevent dropout from vocational education by promoting school connectedness; nevertheless, the dropout rate remained high. Our results point to the need to explore how to further improve the wellbeing at school among young people in vocational education.

**Trials registration:**

ISRCTN, ISRCTN57822968. Registered 16 January 2013 (retrospective registered).

**Electronic supplementary material:**

The online version of this article (10.1186/s40359-018-0258-8) contains supplementary material, which is available to authorized users.

## Background

Education is associated with good health and increased life expectancy [1]. Lower education or lack of formal education may lead to poorer health because of higher occupational risks, more risky health behavior, unemployment and lack of economic resources [2]. There is a clear need to reduce the high dropout rates from vocational education (about 50%) [3]. Vocational education prepares students for immediate entry into the labor market as a skilled worker; as such the education tends to attract students who prefer non-academic learning [4]. From a life-course perspective, dropout is seen as the culmination of a long process of disengagement from school and is associated with poor academic performance and adverse socioeconomic conditions [5, 6]; factors that might be hard to modify. Structural interventions targeting the social processes that take place within school offer a promising way to increase the completion of education [5, 7].

Settings-based health promotion is based on the idea that changes in people’s health and behavior are easier to achieve by focusing on the organizational culture, instead of directly on individuals [8]. Such an approach presents an opportunity to reach all students through their everyday life at school by improving their circumstances and the immediate determinants of dropout [9]. The effect of settings-based interventions in upper-secondary vocational educations remains to be evaluated. Two systematic reviews have shown that improvement of the social environment at school has beneficial effects on school connectedness [10] and dropout [11]; neither reviews investigated the effect on students above the age of 16. A review that included high school students in older ages reported mixed effects, and the authors called for multicomponent interventions that address the school’s organizational structure [7]. Schools can use strategies that develop positive social relations which may enhance participation in educational activities and commitment to school [12–14]. E.g., in their study of Dutch upper-secondary vocational education, Elffers et al. [15] found that good relationships with classmates enhance the students’ sense of belonging to school. In Tinto’s model of college dropout, both academic integration and social integration are major predictors of dropout [16]. Academic and social integration result from students’ interaction with the various dimensions of the school setting: teachers, classmates, institutional climate, and the curriculum [16]. In schools offering vocational education, cigarette smoking can be an influential aspect of the social environment for two reasons: the smoking prevalence is particularly high [17–19] and young people use smoking to socialize and to gain acceptance from fellow students [20]. However, the peer group processes around smoking may diminish the students’ focus on the accomplishment of professional skills, in turn leading to problems in passing the final examination [21].

Shaping the Social was a settings-based intervention aiming to strengthen students’ social relations and increase participation in educational activities with the overall purpose of reducing dropout from vocational education [21, 22]. The intervention program included components centered on improving the way schools welcome new students and components centered on enhancements of student participation in everyday school life by integrating social and educational activities. The latter included class meetings every morning, break policy and a pleasant physical environment.

### Aim

The primary aim of this paper was to examine the effect of the Shaping the Social intervention on school dropout during a 2 year follow-up period. As a secondary aim, we examined how wellbeing at school may impact school dropout. We hypothesized that students in the intervention group would report better wellbeing at school than the control group, and that higher perceived wellbeing at school would reduce the risk of dropping out of school. Therefore, as an exploratory aim, we explored whether there was any evidence to suggest that an intervention effect on dropout was mediated through improved wellbeing at school.

## Methods

### Setting

In the Danish educational system, young people from the age of 16 can choose to continue from compulsory school into upper secondary education; either general education (high school) or vocational education. Almost half of every youth cohort starts in a vocational program, some after being enrolled in a high school. The vocational education is initiated by a basic course with duration of between 20 and 60 weeks and followed by a main program that generally takes about 3 years and require an apprenticeship agreement.

### Study design

A non-randomized controlled trial was used including four intervention and six matched-control schools. In 2009 and 2010, four large vocational schools in urban areas distributed across Denmark were involved in developing the intervention. Inclusion criteria were vocational schools that offered a wide variety of educational programs and which were willing to participate in the development of the intervention. When the final program was presented to the school management, they enrolled more than twice as many departments as had participated in the development phase. Characteristics of the intervention schools were used to select the control schools. Control schools were matched to intervention schools with regard to large school size (≥800 students), urban/suburban location and basic courses within construction, electricity, information technology, auto mechanic, media production, or agriculture. Sixteen schools were eligible, from which eight control schools were selected on the basis of geographic diversity. Of these eight schools, six agreed to participate as control school. One school withdrew due to low resources, and another due to participation in too many projects.

We chose the non-randomized design for two main reasons: (i) There were only 46 technical or agricultural vocational schools in Denmark with substantial differences in size and educational program and if we had randomized within schools, we considered a carry-over effect to be very likely, and (ii) the schools involved in the development phase expected to become intervention schools**.** Health promotion programs in schools work better if they take a whole-school approach in which schools are involved in developing the program, ensuring that the school’s needs as well as local and evidence-based solutions are incorporated.

The intervention was implemented in basic courses that started between October 2011 and October 2012. Control schools continued with their normal practice. The study design is described further elsewhere [23].

### Participants

#### Register-based data

The student population was identified in the Student Register [24] at Statistics Denmark by: (1) school address, (2) type of vocational cluster and (3) date of school start from 1st October 2011 until 31st October 2012. The Student Register contains individual-level information on all persons registered to education, and data are generated from all educational institutions’ administrative records each year. All residents in Denmark have a unique personal identification number; information within and across years was linked through this. The students were followed during a 2–year period. The reason for the long follow-up period was large variability in the length of basic courses depending on the educational program and the students’ prior qualifications.

#### Survey data

To study students’ wellbeing at school, we invited a part of the total student population for participation in two surveys; during the first week of school (i.e., baseline) and at 10-week follow-up. A web-based teacher survey on implementation was also collected after 10 weeks. We employed 10-week assessments because one basic course (the painter course) only lasted 10 weeks. The students filled out web-based questionnaires in the classroom. Non-respondents received a code to the questionnaire by the postal system, e-mail and Short Message Service (SMS). In the questionnaires, the students were asked for their personal identification number in order to link to register data.

### Shaping the social intervention

The intervention program was developed in collaboration with intervention schools. Several of the intervention components were inspired by best practices which we combined in a multifaceted and comprehensive approach. A few components were optional in order to accommodate the variability in the daily practice and approaches between schools.

The mandatory components included:

(i) Incoming students and their parents (or other relatives) are invited to a preliminary meeting before school starts. At the meeting, a teacher presents the education and a guided tour around the school’s facilities is offered. If possible, an older student is the tour guide.
(ii) Welcoming activities during the first school day, including classrooms prepared for a festive reception, welcome speech, person-to-person introduction, and presentation of the curriculum and course content. During the day products of former students are displayed and the new students begin working on an assignment relevant to their education.
(iii) Comprehensive and updated timetable is delivered to the students to avoid confusion and make them able to organize their day. The timetable must contain a clear description of course, meetings times, room assignments and clothing requirements. Once in the introduction period a teacher goes through the curriculum and timetable in order to raise awareness that absence can be a problem.
(iv) Each morning, students and a teacher gather together in a class meeting at which coffee/tea or, preferably, a light breakfast is served. The program of the day is planned, both for the class and the individual student. Moreover, students and the teacher talk about anything and everything; both related to school and what goes on outside school. The aim is to focus students on activities of the day and facilitate interactions between students as well as between teachers and students.
(v) A break policy that comprises scheduled breaks for all students is implemented. This implies that the entire class takes breaks at the same time and no additional breaks, e.g. small smoke breaks, is allowed. The teachers are made aware not to use the term ‘smoke break’.
(vi) Establishment of a pleasant non-smoking environment in order to create a place for students to gather during breaks, for example setting up table football or a cozy sofa area. This area has to serve as an alternative to the smoking areas.

Moreover, two optional components were included: Monthly events during schools hours that included an educational theme integrated with a social activity; Open workshop outside school hours in which students have access to school facilities and a specialist teacher was present. To provide a common platform for understanding the intervention, we have described the compulsory intervention components in terms of behavior change techniques [25] [see Additional file 1]. The behavior change techniques were fitted retrospectively and not used in the development phase. The rationale is described in detail elsewhere [21]. Due to the nature of the intervention, no blinding was possible in this study.

#### Implementation support

Before implementation of the intervention program, we held one meeting for the school management at each intervention school and one or two meetings for middle managers and teachers. These meetings had focus on how to ease the implementation and when to implement. Furthermore, a pamphlet was provided with instruction on implementation and the rationale of the program. During the implementation process, we had discussions (face-to-face or by telephone) with teachers to focus them on target and progress, including solutions for better implementation.

### Measures

#### School dropout

Dates of dropout or completion from the Student Register [24] were used to identify school dropout within the follow-up period. The variable was dichotomized into those who completed the basic course or were still registered versus those who dropped out.

#### Student wellbeing

Four subscales of student wellbeing were used: school connectedness; student support; teacher relatedness; valuing the profession. The scales were obtained from a Danish version of the Health Behavior in School-aged Children (HBSC) survey [26]. School connectedness, student support and teacher relatedness have demonstrated adequate validity and reliability among 13 to 15-year-old students [27]. Inspired by HBSC items on school engagement, new items were developed for the Shaping the Social study to measure valuing the profession (i.e., I am proud of my profession, I feel that I learn many new things about the profession, I enjoy learning about the profession). Student wellbeing was assessed using 13 items with responses given on a 5-point Likert scale ranging from “strongly agree” to “strongly disagree”. Sum scores for each subscale were obtained and a higher score indicates better wellbeing. The four-factor model was evaluated by confirmatory factor analysis [28]. In the current study, Cronbach’s alphas were 0.78 for valuing the profession and 0.85 for the other subscales.

#### Covariates

We used registers in Statistics Denmark covering information on age, sex, ethnicity, socioeconomic position and prior school dropout [29]. From the Danish Civil Registration System, we obtained information on: age at school start (continuous variable), sex, and ethnicity measured by origin (determined by the listed priority: (1) mother’s country of birth, (2) father’s country of birth, (3) student’s country of birth). Parental income was applied as proxy for socioeconomic position. Information on income was retrieved from the Income Statistics Register in 2011. Parents’ disposable income levels were divided into income quintiles for the all Danish residents above 30 years stratified by sex and age, and highest ranking parental income was obtained. Information on prior dropout from vocational education was taken from the Student Register. Life satisfaction, academic self-efficacy and apprenticeship agreement was assessed using student questionnaire. Life satisfaction was measured by the 0–10 Cantril Ladder scale [30] and dichotomized into: high (6–10) versus low (0–5). Academic self-efficacy was measured by the statement: “I can do the hardest school work if I try” [31], on which a binary variable reflecting agreement was constructed. A variable was constructed reflecting apprenticeship agreement (yes, no—high potential, no—low potential), based on study-specific items: “Do you have an apprenticeship agreement?” and “What is the possibility that you will get an apprenticeship agreement?”

#### Adherence to intervention

Adherence to the intervention was measured by items reflecting each component of the intervention program. We used response options ‘yes’, ‘no’ or ‘do not know’ (categorized into yes versus no/do not know). The morning meeting component was determined with responses to ‘How many days did you or another teacher conduct morning meetings for the class in the preceding week?’ (response options: 0, 1, 2, 3, 4, 5,‘do not know’).

### Statistical analyses

A multilevel logistic regression model was used to estimate the intervention effect on school dropout. We used a two-level model with students at level 1 and teams at level 2, allowing for correlation between students from the same team. The register-based data did not cover information on classes. Consequently, we defined “team” as students entering the same vocational cluster (e.g. construction) in the same term at the same school address. This implied that some classes were in the same team. We identified 49 teams in the intervention arm and 149 teams in the control arm. We adjusted for age, sex, ethnicity, parental income, prior school dropout and type of basic course, to account for potential differences between the intervention and control groups at the study onset [6], and to increase the precision of effect estimates.

There was missing information on parental income or ethnicity for almost 4% of the students. For intention-to-treat (ITT) analysis, we handled the missing covariate data by multiple imputation, performed with 10 imputations. The variables used for the imputation were sex, age, ethnicity, parental income, living arrangement, and prior and current school dropout. A complete case analysis was used for sensitivity analysis. For all models, a 5% statistical significance level was applied. However, statistical significant *p*-values indicate little about the practical significance. A way to understand an intervention effect is offered by the number needed to treat (NNT) method [32], which is an estimate of the number of students that need to be subjected to the intervention for one student to benefit. The NNT was estimated for the school dropout outcome using the absolute risk difference and is given by:

$$ \frac{1}{p\left(\mathrm{intervention}\right)-p\left(\mathrm{control}\right)} $$

where *p* is the proportion of students that did not drop out of school (the improvement).

#### Secondary analyses

First, we estimated how the intervention affected each potential mediator using general linear regression. Secondly, we examined how each mediator was associated with school dropout using logistic regression. Finally, we tested the intervention effect on school dropout through the potential mediators. There has been a growing debate about how best to ascertain and estimate mediation. Previous approaches are strongly influenced by the work of Baron and Kenny [33], where a potential mediator is simply added to the model and the change in the effect of the primary variable is examined. This approach works in the special case of linear effects without interactions, but is fundamentally flawed otherwise. New approaches are based on the argument that the only requirement for mediation is that the indirect effect is significant. Models based on the concept of natural direct and indirect effects are able to handle non-linear models [34, 35]. An example is the inverse probability weighting (IOW) approach that make fewer modeling assumptions. It condenses the association between exposure (i.e. the intervention) and mediators, conditional on covariates, into a weight, removing the need to specify a regression model for regression of the outcome on the exposure and mediator. The weight is used to estimate the natural direct effect in a weighted regression analysis [36]. Practical guidance for conducting mediation analysis using inverse odds ratio weighted estimation approach, including STATA code examples, has been provided by Nguyen et al. [36]. To apply the IOW method, we determined the predicted odds for the intervention from the mediator plus the baseline covariates, obtained in a logistic regression model. Next, we took the inverse of the predicted odds to compute the IOW weights. Total effect on school dropout was estimated using a generalized linear model with a logit link. This analysis was replicated, including the IOW weights, estimating the direct effect by adjusting for the mediator. Ultimately, the indirect effect was calculated by subtracting the direct effect from the total effect. We used bias-corrected bootstrapping (1000 samples) to recover correct standard errors and derive confidence intervals for direct and indirect effects.

The indirect effect (i.e., mediators that explain a possible observed relationship between intervention and school dropout outcome) is identifiable if three assumptions are met: there has to be no unmeasured confounding of (a) the exposure-mediator relation, (b) the exposure-outcome relation, and (c) the mediator-outcome relation [36]. These assumptions follow from standard epidemiological concepts of confounding. To adjust for the potential confounding of the non-randomized design (the first two assumptions), we included baseline age, sex, ethnicity, parental income, prior school dropout and type of basic course. To adjust for the potential confounding of the mediator–outcome relationship, we additionally adjusted for self-reported life satisfaction, academic self-efficacy and apprenticeship agreement measured at baseline [37–39].

Analyses were performed using SAS v9.4 (SAS Institute Inc., Cary, NC) and the mediation analyses were completed by Stata v14.0 (StataCorp LP, College Station, TX).

## Results

### Participant flow and baseline characteristics

**Fig. 1:**
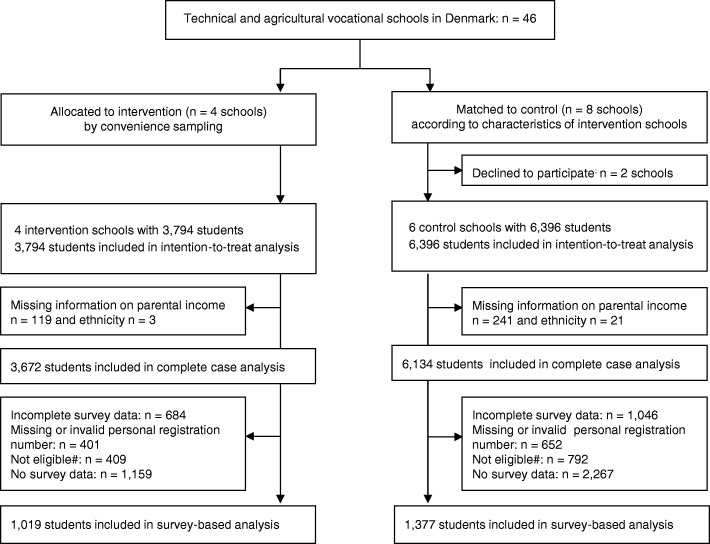
Flow diagram of Shaping the Social. ^#^Dropped out of school before the 10-week survey

A total of 3794 students were registered in intervention schools and 6396 students in control schools (*n =* 10,190) (Fig. 1). The survey sample included 1019 students in the intervention condition and 1377 students in the control condition (*n =* 2396) (Fig. 1).

Of the 10,190 students, mean age was 22 years, and 2984 (29%) had a history of prior school dropout (Table 1). Non-western students and men were under-represented in the intervention group. There was no loss to follow-up on primary outcome (i.e., school dropout). Compared to the total student population, a lower proportion of students in the survey sample were of non-western origin and had previously dropped out of vocational education and a higher proportion were living with parents [see Additional file 2].

**Table 1:** Baseline characteristics of the student population^a^ (*N* = 10,190) by intervention and control

	Intervention	Control
	(*n* = 3794)	(*n* = 6396)
Age (years), mean ± SD	21.8 ± 6.3	21.5 ± 5.6
Men, n (%)	2928 (77)	5357 (84)
Non-western ethnicity, n (%)	206 (5.4)	840 (13)
Living with parents, n (%)	2106 (56)	3567 (56)
Parental income, n (%)		
1 Lowest	306 (8.3)	592 (9.6)
2	645 (18)	1004 (16)
3	830 (23)	1350 (22)
4	1021 (28)	1530 (25)
5 Highest	873 (24)	1679 (27)
Parental education, n (%)		
High	1022 (28)	1939 (32)
Medium	1973 (54)	3058 (50)
Low	648 (18)	1072 (18)
Prior school dropout, n (%)	1128 (30)	1856 (29)

^a^All students who were enrolled at technical or agricultural departments at 4 intervention schools and 6 control schools

### Intervention effect on school dropout

**Fig. 2:**
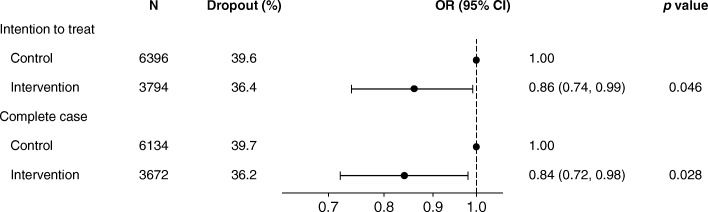
Effect of Shaping the Social on school dropout within 2 years (*n* = 10,190). Adjusted for baseline age, gender, ethnicity, parental income, prior school dropout, type of basic course and teams (random effect)

At 2-year follow-up, the dropout rates were 36% in the intervention group and 40% in the control group (Fig. 2), corresponding to number needed to treat (NNT) of 31. The intention to treat analysis (ITT) showed that intervention students had an odds ratio (OR) of 0.86 (confidence interval (CI): 0.74, 0.99; *p* = 0.046) for dropout compared to control students. The complete case analysis produced similar results to the ITT analysis (OR = 0.84, 95% CI: 0.72, 0.98; *p* = 0.028).

When examining the intervention effect measured at 6, 9, 12, and 18-month follow-up, respectively, the odds ratios were similar to the 2-year assessment. The magnitude of difference in dropout between the intervention and control group though increased (e.g., at 6 month school dropout rates in intervention and control groups was 24% and 26%, respectively, as shown in Additional file 3).

### Intervention effect on school dropout mediated through student wellbeing

**Fig. 3:**
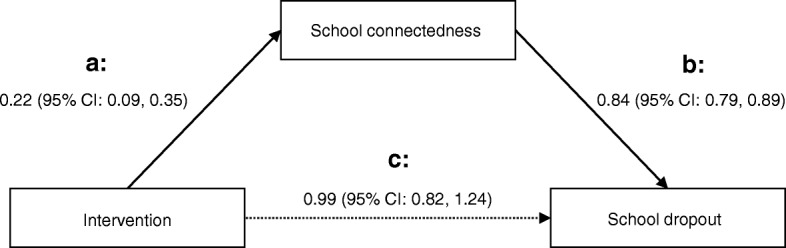
School connectedness as a mediator of the intervention effect on school dropout (*n* = 2396). The two solid arrows represent the indirect effect of the intervention on school dropout through school connectedness, and the dashed arrow represents the direct effect after adjustment of school connectedness. **a** The school connectedness score was 0.22 units higher in intervention group compared to control group. **b** For one unit increase in school connectedness score the odds ratio for dropout was 0.84. The odds ratio for intervention effect on dropout through school connectedness was 0.92 (95% CI: 0.85, 0.99), *p* = 0.032 (indirect effect; see Table 2). **c** There was no intervention effect that did not go through school connectedness (OR = 0.99, 95% CI: 0.82-1.24)

At 10-week follow-up, students in the intervention group showed higher mean scores for school connectedness (*p* < 0.01) and valuing the profession (*p* < 0.05) than students in the control group (Table 2). The odds ratio for the effect that the intervention had on school dropout through school connectedness was 0.92 (95% CI: 0.85, 0.99), *p* < 0.05). The mediation analysis did not identify any effect of the intervention on dropout beyond the effect mediated via school connectedness (OR = 0.99, 95% CI: 0.82, 1.24) (Fig. 3).

**Table 2:** Intervention effect on mediators, mediators’ effect on dropout, and intervention effect on dropout through mediators (*N* = 2396)

	Intervention effect on mediator	Mediator effect on dropout	Intervention effect on dropout through mediator
	Mean difference (95% CI)^a^	OR (95% CI)^ab^	OR (95% CI)^a^
School connectedness	0.22 (0.09, 0.35)^**^	0.84 (0.79, 0.89)^***^	0.92 (0.85, 0.99)^*^
Student support	0.19 (− 0.07, 0.44)	0.95 (0.93, 0.98)^**^	0.95 (0.88, 1.03)
Teacher relatedness	0.07 (− 0.10, 0.24)	0.91 (0.87, 0.95)^***^	0.96 (0.88, 1.03)
Valuing the profession	0.17 (0.02, 0.31)^*^	0.82 (0.78, 0.87)^***^	0.95 (0.88, 1.03)

^*^ *p* < 0.05; ^**^ *p* < 0.01; ^***^ *p* < 0.001

^a^Adjusted for baseline age, sex, ethnicity, parental income, prior school dropout, life satisfaction, academic self-efficacy, apprenticeship agreement

^b^Adjusted for intervention condition

### Effects of student wellbeing on school dropout

Higher levels of school connectedness, student support, teacher relatedness, and valuing the profession were all associated with reduced school dropout (Table 2). In particular, the dropout rate was reduced by higher units of school connectedness (OR = 0.84, 95% CI: 0.79, 0.89) and valuing the profession (OR = 0.82, 95% CI: 0.78, 0.87).

### Adherence to intervention

The adherence to Shaping the Social was highest for introduction activities; 97% had prepared the classroom for a festive reception on the first school day and presented the curriculum and course content for the new students (Table 3). The adherence was lowest for break policy; 38% of the intervention classes complied with the break policy.

**Table 3:** Teacher-reported implementation degree

	Number of classes	Implemented, n (%)
*1. Meeting before school start*		
A preliminary meeting was held	73	63 (86%)
Relatives invited	62	56 (90%)
Guided tour around the school’s facilities	63	36 (57%)
Gathered in educational tracks	51	40 (78%)
*2. Welcoming at first school day*		
Classroom prepared for a festive reception	72	70 (97%)
Welcome speech	72	59 (82%)
A round of person-to-person introductions	72	57 (79%)
Students work in groups on an assignment relevant to the education	72	45 (63%)
Display of products of former students	72	46 (64%)
Presentation of the curriculum and course content	71	69 (97%)
Plan for the day, so others can take over	72	57 (79%)
*3. Clear and detailed timetable*		
Clear description of time	72	67 (93%)
Clear description of classrooms’ location	72	62 (86%)
*4. Morning meetings every school day*		
Class meetings (number of days per week):		
5	72	26 (36%)
4	72	7 (10%)
3	72	10 (14%)
2	72	6 (8%)
1	72	10 (14%)
0	72	13 (18%)
Beverage or food served	59	29 (49%)
*5. Break policy*		
Entire class taking breaks at the same time and smoke breaks not allowed	72	27 (38%)
*6. Pleasant non-smoking place to gather during breaks*		
Existence of a pleasant non-smoking place (e.g. table football)	72	43 (60%)

Table reproduced from published article regarding proximal effects [28]

## Discussion

We found that Shaping the Social students were less likely than control students to drop out from vocational education. Our results indicate that the intervention effect was mediated through school connectedness. Moreover, we demonstrated that the risk of dropping out decreased with improved student wellbeing, i.e. school connectedness, student support, teacher relatedness and valuing the profession; however, no intervention effects were found for student support, teacher relatedness, or valuing the profession.

Public health significance is not easily translated into clinical or personal significance. However, we estimated that the number needed to treat was 31, meaning that, on average, 31 students must be exposed to Shaping the Social to prevent one student from dropping out. In the regular vocational classes (i.e. control classes) 40% drop out which equals 12 of 31 students; helping one out of 12 students to succeed in the educational system seems significant. A meta-analysis of dropout interventions in high schools found an average eight percentage point reduction in dropout between intervention programs and regular educational programs [40]. In our study, we found a four percentage point difference. The interventions included in the meta-analysis occurred over a long time, about two school years, while the current study averaged 5 months (i.e., the duration of the basic courses), which might account for some of the difference.

As with comparable interventions conducted among a younger student population [10], we found that Shaping the Social had positive impact on increasing school connectedness. The lack of effects from the other mediators might be due to sensitivity and intensity. Provided that social support is the product of relationships that develop and change slowly, significant effects may not be found until longer-term follow-up; in this study we measured wellbeing at week 10. Secondly, there might be measurement issues relating to the items used to capture the wellbeing factors. Finally, the intervention might not have been intensive enough to create an impact on social support. Low implementation is a well-known problem in school-based interventions [11]. Public health interventions work through social processes and, in our case, the implementation depended on the readiness of the teachers [41]. Data from the study indicated that restructuring the daily school practices might be a harder task than implementing new practices regarding how to welcome new students. For example, only 36% of classes had daily morning meetings whereas the majority of classes had implemented the introduction activities. The fact that the introduction activities seemed easier to implement may explain the effects of our study, given that a welcoming environment might be a major factor for promoting school connectedness [42] and preventing dropout [43].

The finding that student wellbeing was related to school dropout, independently of the intervention, underscores the importance of the school environment for vocational students. This association is well-established among younger students [44]; our study showed that particular school connectedness and valuing the profession developed during the first few months of school were strong determinants for completing the education.

Strengths of the present study included the use of register-based data which led to the obtainment of objective measures and inclusion of the entire student population. Therefore, misclassification of the outcome and risk of attrition bias were avoided. Furthermore, the intervention was carefully developed in order to fit to the setting [45], however evaluating programs anchored in an ecological approach is a challenge [46]. A way of dealing with its complexity is unpacking the theory of change [47]. As such, we examined the associations between the intervention, potential mediators and school dropout. Students who had already dropped out of school were not included in the questionnaire subsample, thus change in the mediator preceded change in the outcome as required for establishing a causal relation [48]. Moreover, we tested whether the student wellbeing outcomes were predictive of school dropout (independently of intervention) which is a way to validate the theoretical construction of the program theory and can inform future intervention developers about which determinants to target [47, 49].

There are a number of notable limitations of this study. The selected schools were not randomly assigned, leading to potential selection bias. Random allocation of intervention and control schools was not feasible due to the heterogeneous nature and a limited number of Danish vocational schools. Additionally, randomization was not a logical choice; it was natural for the schools that took part in the development of the intervention program to apply it and we hypothesized that it will make the intervention program work better [50]. To avoid selection bias, control schools were selected to be minimally different from the intervention group, and the statistical analyses were controlled for potential confounders. Still, it is possible that important covariates were omitted and unobserved confounding may have occurred. Interestingly, the meta-analysis by Wilson and colleagues [11] demonstrated that randomized and non-randomized studies of dropout prevention programs had equivalent effect sizes.

The survey sample precluded generalizations of our results regarding student wellbeing to students who dropped out during the first 10 weeks of school. Additionally, student wellbeing was assessed by self-report. Self-report will always be an issue when using questionnaire-based data. Although the students were guaranteed confidentiality and informed of the exclusion of identification, social desirability bias may have occurred. However, it is likely that such a bias is non-differential, because the students were probably not aware of participating in an intervention study.

The trial registration was done retrospectively rather than prospectively. Prospective trial registration reduces the temptation to either not publish or only publish selective results from completed trials [51]. Our reason for the retrospective registration was lack of awareness; however the registration was still done during the data collection process and before the data analysis.

## Conclusions

Our study suggested that Shaping the Social was effective in reducing dropout for vocational school students; however the dropout rate remained high in the intervention group. The intervention effect was mediated through students’ feeling of being connected to their school; however independently of the intervention both school connectedness, student support, teacher relatedness and valuing the profession were identified as important factors in preventing dropout. Improving the school environment should be a central part of preventing dropout from vocational school, thus more research to explore how to further develop positive peer relationships and teacher-student relationships is warranted. Additionally, future research should also look at how to make implementation feasible within the existing organizational challenges. Making significant changes to everyday school life at a heterogeneous educational organization, as the Danish vocational school system represent, requires that school managers are continually supporting the teachers by delivering resources (e.g. time and information) and take part in regular meetings at which clarifying questions and disputed points are discussed.

## Additional files

Additional file 1 Identifying Shaping the Social intervention content using the behavior change techniques (BCT) taxonomy (v1) and linked to the theoretical determinants of behavior change (TDF). (DOCX 23 kb)

Additional file 2 Baseline characteristics of students in survey sample (*N* = 2396), by intervention and control groups. (DOCX 20 kb)

Additional file 3 Odds ratios (OR) for school dropout at 6, 9, 12, 18 and 24 month follow-up in intervention group compared to control group. Odds ratios estimated from complete case analysis adjusted for age, sex ethnicity, parental income, prior school dropout, type of basic course and classes (random effect). *N* = 9652. (DOCX 28 kb)

## Abbreviations

CI: Confidence interval
ICC: Intra class correlation coefficient
IOW: Inverse odds weighting
ITT: Intention to treat
N: Number
OR: Odds ratio
SD: Standard deviation
