# Supplementary material for: Effectiveness of the settings-based intervention Shaping the Social on preventing dropout from vocational education: a Danish non-randomized controlled trial
Source: BMC Psychol. 2018 Sep 12;6:45. doi: 10.1186/s40359-018-0258-8 (PMC6134754; doi:10.1186/s40359-018-0258-8)
Supplement: Supplementary file 1 — Identifying Shaping the Social intervention content using the behavior change techniques (BCT) taxonomy (v1) and linked to the theoretical determinants of behavior change (TDF). (DOCX 23 kb) [file 40359_2018_258_MOESM1_ESM.docx]

**Additional file 1** Identifying Shaping the Social intervention content using the behavior change techniques (BCT) taxonomy (v1) and linked to the theoretical determinants of behavior change (TDF).

|  |  | **BCTs** |  | **TDF** | **Rationale** |
| --- | --- | --- | --- | --- | --- |
| **Preliminary meeting at the school** |  |  |  |  |  |
| A guided tour around the school and its facilities.  A description of the profession.  Presentation of products from older students. |  | Information about social and environmental consequences, social support (unspecified), social support (practical) |  | Knowledge, optimism, social influences | Students know locations of classrooms and get positive impression of the school and the education. Parents meet teachers. |
| **Welcoming at first school day** |  |  |  |  |  |
| Classrooms prepared for a festive reception, welcome speech and person-to-person introduction. The protocol is updated with all students' names within each class. Plan for the day so others can take over in case of e.g. illness. Presentation of the curriculum and content of the basic course.  Display of products of former students. Group work on an assignment relevant for the education. |  | Restructuring the social environment, social support (unspecified)  Instruction on how to perform a behavior  Demonstration of the behavior |  | Social influences, optimism, knowledge | Students feel welcome, and get to know each other.  Students experience that the school is on top of the situation.  Students know what they will be taught in the course.  Students experience what they will be taught in the course. |
| **Comprehensive timetable** | | |  |  |  |
| A sufficient timetable with clear descriptions of course, time and clothing requirements. |  | Prompts/cues |  | Knowledge, reinforcement | Students know where to attend and what to wear, and able to plan and organize their day. |
| **Morning meeting at class level** |  |  |  |  |  |
| Coffee/tea is served, preferably a light breakfast meal.  The program of the day is planned, both for the class and the individual student.  Students and teachers talk about anything and everything – related to school and outside school. |  | Restructuring the social environment, social support (unspecified) |  | Social influences, optimism | Students gather around a joint social activity. The student-student and student-teacher relation is strengthened.  Students know what to do during the day. |
| **Scheduled breaks** |  |  |  |  |  |
| Entire class takes breaks at the same time.  Smoking is only allowed during these breaks. No use of the term 'smoke break'. |  | Restructuring the social environment, avoidance and reducing exposure to cues for the behavior |  | Social influences, optimism | Students can concentrate during lessons. Smokers and non-smokers have the same working conditions. Focus on activities other than smoking. |
| **Pleasant non-smoking area** |  |  |  |  |  |
| Setting up of an area in order to create a place for hanging out. For example, seating arrangement, coffee machine, table football. |  | Restructuring the physical and social environment, adding objects to the environment |  | Social influences, environmental context and resources | Students know where to go to hang out with others, and have a common alternative to the smoking areas. |
| **Materials provided to aid implementation** | | |  |  |  |
| Pamphlet provided instruction on implementation. Discussion on ease of implementation and how/when to implement |  | Verbal persuasion about capability, instruction on how to perform behavior |  | Knowledge, beliefs about capabilities | To make implementation more convenient |
| **Ongoing audit and feedback to teachers** | | |  |  |  |
| Generation of solutions for better implementation. Discussion of improvements made. |  | Problem solving, action planning, social reward |  | Goals, beliefs about capabilities, reinforcement, | To focus teachers on target and progress |

BCT = Behavior Change Techniques; TDF = Theoretical Domains Framework (i.e., why the techniques work).
