# Supplementary material for: Effectiveness of the settings-based intervention Shaping the Social on preventing dropout from vocational education: a Danish non-randomized controlled trial
Source: BMC Psychol. 2018 Sep 12;6:45. doi: 10.1186/s40359-018-0258-8 (PMC6134754; doi:10.1186/s40359-018-0258-8)
Supplement: Supplementary file 2 — Baseline characteristics of students in survey sample (N = 2396), by intervention and control groups. (DOCX 20 kb) [file 40359_2018_258_MOESM2_ESM.docx]

**Additional file 2** Baseline characteristics of students in survey sample (N=2,396), by intervention and control groups.

|  |  | Intervention | Control |
| --- | --- | --- | --- |
|  |  | (n=1,019) | (n=1,377) |
| Age (years, mean ±SD) |  | 21.0 ± 5.6 | 20.4 ± 5.3 |
| Men, n (%) |  | 716 (70) | 1,180 (86) |
| Non-western ethnicity, n (%) |  | 23 (2.3) | 102 (7.4) |
| Living with parents, n (%) |  | 646 (63) | 932 (68) |
| Parental income, n (%) |  |  |  |
| 1 Lowest |  | 70 (6.9) | 103 (7.5) |
| 2 |  | 143 (14) | 210 (15) |
| 3 |  | 247 (24) | 303 (22) |
| 4 |  | 300 (29) | 383 (28) |
| 5 Highest |  | 259 (25) | 378 (27) |
| Parental education, n (%) |  |  |  |
| High |  | 297 (29) | 390 (29) |
| Medium |  | 554 (55) | 771 (57) |
| Low |  | 162 (16) | 191 (14) |
| Prior school dropout, n (%) |  | 211 (21) | 272 (20) |
| High academic self-efficacy, n (%) |  | 536 (51) | 735 (52) |
| High life satisfaction, n (%) |  | 874 (86) | 1,174 (86) |
| Apprenticeship agreement, n (%) |  |  |  |
| Yes |  | 123 (12) | 248 (18) |
| No, but high potential |  | 437 (43) | 570 (42) |
| No, low potential |  | 456 (45) | 554 (40) |

Abbreviations: n = number of students, SD = standard deviation.
