# Supplementary material for: Effectiveness of the settings-based intervention Shaping the Social on preventing dropout from vocational education: a Danish non-randomized controlled trial
Source: BMC Psychol. 2018 Sep 12;6:45. doi: 10.1186/s40359-018-0258-8 (PMC6134754; doi:10.1186/s40359-018-0258-8)
Supplement: Supplementary file 3 — Odds ratios (OR) for school dropout at 6, 9, 12, 18 and 24 month follow-up in intervention group compared to control group. Odds ratios estimated from complete case analysis adjusted for age, sex ethnicity, parental income, prior school dropout, type of basic course and classes (random effect). N = 9652. (DOCX 28 kb) [file 40359_2018_258_MOESM3_ESM.docx]

OR=0.85

(95% CI: 0.73,0.99)

OR=0.85

(95% CI: 0.74,0.99)

OR=0.86

(95% CI: 0.74,0.99)

OR=0.84

(95% CI: 0.72,0.98)

OR=0.86

(95% CI: 0.74,0.99)

NNT = 31

**Additional file 3** Odds ratios (OR) for school dropout at 6, 9, 12, 18 and 24 month follow-up in intervention group compared to control group. Odds ratios estimated from complete case analysis adjusted for age, sex ethnicity, parental income, prior school dropout, type of basic course and classes (random effect). N = 9,652.
